# Fixation Patterns of Chinese Participants while Identifying Facial Expressions on Chinese Faces

**DOI:** 10.3389/fpsyg.2017.00581

**Published:** 2017-04-12

**Authors:** Mu Xia, Xueliu Li, Haiqing Zhong, Hong Li

**Affiliations:** ^1^College of Ethnology and Sociology, Guangxi University for NationalitiesNanning, China; ^2^Sports Institute, Guangxi Teachers Education UniversityNanning, China; ^3^College of Psychology and Sociology, Shenzhen UniversityShenzhen, China

**Keywords:** Chinese participants, fixation model, upper part of the face, lower part of face, etiquette culture

## Abstract

Two experiments in this study were designed to explore a model of Chinese fixation with four types of native facial expressions—happy, peaceful, sad, and angry. In both experiments, participants performed an emotion recognition task while their behaviors and eye movements were recorded. Experiment 1 (24 participants, 12 men) demonstrated that both eye fixations and durations were lower for the upper part of the face than for the lower part of the face for all four types of facial expression. Experiment 2 (20 participants, 6 men) repeated this finding and excluded the disturbance of fixation point. These results indicate that Chinese participants demonstrated a superiority effect for the lower part of face while interpreting facial expressions, possibly due to the influence of eastern etiquette culture.

## Introduction

Cultural differences between Western and Eastern societies have been shown in many aspects of people’s behavior and cognitions. For example, evidence from subjective well-being research showed that Eastern societies are more likely to hold dialectical emotional styles compared to Western societies. In other words, people in Eastern societies are more likely to view positive and negative emotions as compatible, whereas those in Western societies are more likely to view them as in conflict with each other. These differences are perhaps due to differences in Eastern and Western philosophies (Schimmack et al., 2002). Similarly, attention studies have found that Eastern pay more attention to backgrounds during visual tasks, which enables the understanding of relationships between objects and changes, while Westerners pay more attention to the target, which enables understanding of the features of an object (Liu et al., 2013). McCarthy et al. (2006) suggested that when Westerners know the answer to a question, they tend to maintain eye contact, while Easterners were more evasive. In addition, when searching for the answer to a question, Westerners often look toward the sky, while Easterners look toward the ground.

Facial information, which conveys one’s racial identity, gender, and facial expressions, is very important to human socialization. As humans are social creatures, the accurate interpretation of another person’s facial information would help us to better navigate the social environment. Eye movement patterns when recognizing a face has been shown to be subject to cultural differences. For example, Blais et al. (2008) found that Western Caucasian participants reproduced a scattered triangular pattern of fixations for faces of both races and across tasks. On the Contrary, East Asian participants focused more on the central region of the face. Caldara et al. (2010) found that in a natural situation, Westerners paid more attention to the eyes when looking at an emotionally neutral face, while Chinese paid more attention to the nose. Those evidence suggests that there may be cultural differences in eye fixation patterns when gazing at a face.

Indeed, in Western culture, direct eye contact with others while communicating is usually encouraged (Argyle and Cook, 1976). Thus, due to cultural influences, the eyes may be more important than the nose and mouth for facial expression recognition in Western culture. Many studies that used Western participants and Western faces as stimuli support this view. For example, Vassallo et al. (2009) found that Western participants had more fixations and longer fixation durations on the eyes, in comparison to the nose and mouth, when attempting to identify emotions from faces. Additionally, evidence from the eye movements of Western individuals with social interaction problems, such as autism or social phobia, indicates that these individuals do not gaze directly at the eyes when asked to identify the facial expressions of others (Horley et al., 2003, 2004; Senju and Johnson, 2009a). This phenomenon, called “avoidance of eye contact,” may impair social behavior abilities. However, this phenomenon does not mean these individuals lack social skills—it could be a self-defense mechanism for avoiding anxious experiences, possibly due to negative critical statements given by others in the past, which is automatically activated when these individuals make eye contact with others (Schneier et al., 2011). This evidence indirectly confirms the importance of information in the eyes for recognizing facial expressions in Western cultures. Researchers proposed “the eye contact effect” to explain the benefit of looking at the eyes to infer emotional information, positing that making contact with others’ eyes could modulate recognition and activate the part of the social brain responsible for processing emotion (Emery, 2000; Senju and Johnson, 2009b). Thus, the eyes are critical for recognizing facial expressions in Western cultures.

In contrast, in Eastern, affected by the etiquette culture (one aspect of Confucianism), direct eye contact is considered rude and should be avoided while communicating (Argyle and Cook, 1976). This raises the question of whether individuals from Eastern cultures develop a different pattern from those from Western cultures when identifying facial expressions due to influences from their culture. This could explain why some people view the lower part of the face (nose and mouth) more than the eyes. Jack et al. (2009) found that people from Eastern cultures use more information from the upper part of a face to recognize a facial expression, including happy and neutral faces, which was not consistent with previous research (Cui et al., 2009; Caldara et al., 2010). In addition, Tan et al. (2012) found that rather than adopting the Eastern or Western fixation pattern, Malaysian Chinese participants use a mixed strategy by focusing on the eyes and nose more than the mouth. Thus, this inconsistency in previous studies requires further investigation using eye-tracking methodology.

We devised two experiments to test this question. Experiment 1 investigated whether Chinese participants developed a pattern of avoiding eye contact when identifying facial expressions displayed by Eastern faces. Experiment 2 which base on the outcome of Experiment 1, investigated whether this pattern due to the guidance of the first fixation point.

## Experiment 1

### Participants

Twenty four healthy Chinese undergraduates of Han origin (12 men) participated in this experiment. Participants’ mean age was 20 ± 1.4 years. All had normal or corrected-to-normal vision and none were aware of the purpose of the study. This study (for both Experiments 1 and 2) was carried out in accordance with the recommendations of IRB (the institutional review board) at Guangxi University for Nationalities with written informed consent from all subjects. All subjects gave written informed consent in accordance with the Declaration of Helsinki. The protocol was also approved by the IRB at Guangxi University for Nationalities.

### Apparatus and Stimuli

Four types of facial expressions (happy, peaceful, sad, and angry) were selected from a set of grayscale photographs created by the Chinese Affective Picture System (CAPS) (Wang and Luo, 2005). Forty pictures were used for the formal experiment; each emotion was represented by 10 pictures. In addition, we chose 20 other facial expression pictures, four for use in the classic learning phase and 16 for the practice phase. Each picture measured 1280 × 1024 pixels. Before the experiment, each facial expression was aligned in terms of the position of the eyes, nose, and mouth, and matched in illumination and contrast.

The experiment was conducted in an isolated and quiet room. The participants were asked to sit approximately 55 cm away from a 19-inch CRT display monitor with a sample rate of 85 Hz, and to place their fingers on a keyboard using the standard keyboard fingering. Their heads were fixed on a “U” frame. Eye movements were recorded with an EyeLink tracker (EyeLink II, SR Research^®^, Toronto) with a 250-Hz temporal resolution and a 0.20° spatial resolution.

### Procedure

**FIGURE 1:**
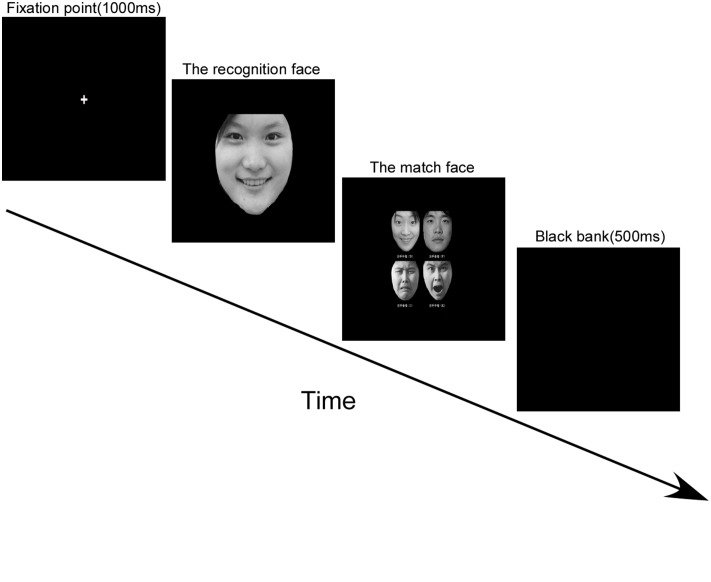
**The procedure for Experiment 1**.

All experiment codes were run on E-prime 1.1. The experimental session was based on the research of Hampson et al. (2006), which consisted of four steps: introduction, classic sample learning, practice, and formal experiment. After the participants viewed the introduction and indicated their understanding of the task, they proceeded to the classic sample-learning step. Four facial expressions—happy, peaceful, sad, and angry, in that order—were shown at the center of the screen with a sentence in Chinese, “This is happy/peaceful/sad/angry,” simultaneously displayed across the top of the screen. Participants were asked to press the spacebar when they recognized each facial expression. The aim of this step was to allow the participant to be acquainted with the classic model of these four types of facial expressions. The next step was practice (**Figure 1**). A trial started with a fixation point appearing in the middle of the screen for 1000 ms, followed by one of the four types of emotion faces (the recognition face), in random order, displayed at the center of the screen. Participants were asked to identify the emotion and press the spacebar as quickly as possible. After this, four smaller emotion faces, previously shown in the classic sample-learning phase, were displayed. The participants’ task was to choose the face (the match face) that best matched the recognition face shown previously. At the same time, four cues written in Chinese, which referred to the four emotions, were displayed beneath the corresponding pictures, so that participants did not need to remember which finger corresponded to which emotion. These cues read: “D (left middle finger) is for happy, F (left index finger) is for peaceful, J (right middle finger) is for sad, and K (right index finger) is for angry.” Because the individuals in the recognition photographs were different from those in the match face photographs, participants could not respond correctly by making a direct identity feature match—a definitive emotional code for the recognition face was required. The participants received feedback after each response. The trial ended with a 500-ms blank screen. To ensure highly accurate data, only the participants with accuracy rates (AC) of 80% or higher advanced to the formal experiment. After practice, each participant was given a 13-point calibration procedure before proceeding to the formal experiment. This procedure was the same as that for the practice except that there was no feedback. The EyeLink tracker recorded the eye movement data associated with participants’ attempts to identify a facial expression only in the formal experiment.

### Defining AOI (Area of Interested)

The way to define AOI in this research was to draw a boundary halfway between the brows and philtrum, separating the face into eye (upper half of the face) and non-eye (lower half of the face) AOIs.

### Results

#### Accuracy According to Facial Expression

The detail data for Accuracy (AC) in Experiment 1 was recorded in the **Table 1**. We used ANOVA to test if there were significant differences in AC, with emotion type as the within-subjects factor. The results showed a significant main effect of emotion type, *F*(3,92) = 3.936, *p* < 0.05, *M* = 1.00, *SD* = 0 for happy face; *M* = 0.95, *SD* = 0.11 for pace face; *M* = 0.91, *SD* = 0.11 for sad face and *M* = 0.95, *SD* = 0.08 for anger face. A least significant difference (LSD) comparison found that the AC of happy were higher than the sad (*p* < 0.01), no other significant differences were found.

**Table 1:** Mean (SD) of accuracy for the four emotion faces in Experiments 1 and 2.

	Happy	Peaceful	Sad	Angry
Experiment 1 (*n* = 24)	1.00 (0.00)	0.95 (0.11)	0.91 (0.11)	0.95 (0.08)
Experiment 2 (*n* = 20)	0.99 (0.01)	0.98 (0.01)	0.90 (0.02)	0.85 (0.03)


#### AOI Analysis

Using the described AOIs, the statistical analysis of the fixation numbers and durations in different AOIs was conducted, only the right trails were analyzed.

The detail data for fixation in Experiment 1 was presented in the **Table 2**. We used repeated measures ANOVA to test if there was a significant difference in the number of fixations, with type of emotion expressed and AOI as within-subject factors. The main effect of emotion types is not significant, *F*(3,57) = 2.087, *p* > 0.05. The main effect of AOI was significant, *F*(1,19) = 52.494, *p* < 0.0001, *M* = 0.71, *SD* = 0.79 for eye AOI and *M* = 3.65, *SD* = 1.28 for non-eye AOI, indicating that the number of fixations for the eye AOI was lower than that for the non-eye AOI. The interaction between emotion types and AOI was not significant, *F*(3,57) = 0.299, *p* > 0.05.

**Table 2:** Mean (SD) of fixation for the four emotion faces in Experiments 1 and 2.

	Experiment 1 (*n* = 24)		Experiment 2 (*n* = 20)	
		
Face type	Eyes AOI	Non-eye AOI	Eyes AOI	Non-eye AOI
Happy	0.49 (0.61)	3.55 (1.46)	0.33 (0.52)	3.53 (0.99)
Peace	0.79 (1.11)	3.74 (1.55)	0.76 (1.19)	3.74 (1.33)
Sad	0.88 (1.01)	4.00 (1.60)	0.74 (0.95)	4.27 (1.66)
Anger	0.69 (0.85)	3.48 (1.68)	0.94 (0.98)	4.67 (1.79)


The detail data for duration in Experiment 1 was presented in the **Table 3**. We used repeated measures ANOVA to test if there was a significant difference in fixation duration, with emotion type and AOI as the within-subject factors. The main effect of emotion types is not significant, *F*(3,63) = 2.537, *p* > 0.05. The main effect of AOI was significant, *F*(1,21) = 36.834, *p* < 0.0001, *M* = 175.00, *SD* = 156.52 for eye AOI and *M* = 940.96, *SD* = 523.90 for non-eye AOI, the fixation duration on the upper face was shorter than that of the lower face. The interaction between emotion type and AOI was significant, *F*(3,63) = 3.324, *p* < 0.05. The simple effect demonstrated that the duration of lower face of happy (*M* = 915.54, *SD* = 542.41), pace (*M* = 980.34, *SD* = 525.73), and anger (*M* = 882.52, *SD* = 648.59) is shorter than sad (*M* = 1062.77, *SD* = 531.20).

**Table 3:** Mean (SD) of duration for the four emotion faces in Experiments 1 and 2.

	Experiment 1 (*n* = 24)		Experiment 2 (*n* = 20)	
		
Face type	Eyes AOI	Non-eye AOI	Eyes AOI	Non-eye AOI
Happy	107.16 (170.42)	915.54 (542.41)	63.27 (92.81)	887.94 (295.70)
Peace	147.29 (174.91)	980.34 (525.73)	279.40 (349.54)	907.81 (222.91)
Sad	183.87 (202.92)	1062.77 (531.20)	228.15 (290.36)	1133.64 (376.39)
Anger	207.02 (236.83)	882.52 (648.59)	280.61 (294.95)	1067.46 (421.88)


The most important result in Experiment 1 is that the number of fixations and durations for Chinese participants were less for the upper part of the face (eyes AOI) compared to the lower part of the face (non- eye AOI) to all four types of facial expressions. This opposite pattern compared to those of studies using Western samples (Vassallo et al., 2009) may due to the Chinese special etiquette culture. However, there may be another explanation. The position of the fixation point we used to help participant to focus their attention in the beginning of a trail was on middle of the screen. The faces were also presented on the middle of the screen, but the areas for the lower part of a face (non-eye AOI) were normally bigger than the upper ones (eyes AOI). This may put the position of the fixation point into the lower part of a face and thus guide participants to pay their attention on it. For exclude this possibility, we then designed Experiment 2.

## Experiment 2

### Participants

Twenty healthy Chinese undergraduates of Han origin (six men) participated in this experiment. Participants’ mean age was 21 ± 1.4 years. All had normal or corrected-to-normal vision and none were aware of the purpose of the study.

### Apparatus and Stimuli

All apparatus and stimuli in Experiment 2 were the same as Experiment 1.

### Procedure

**FIGURE 2:**
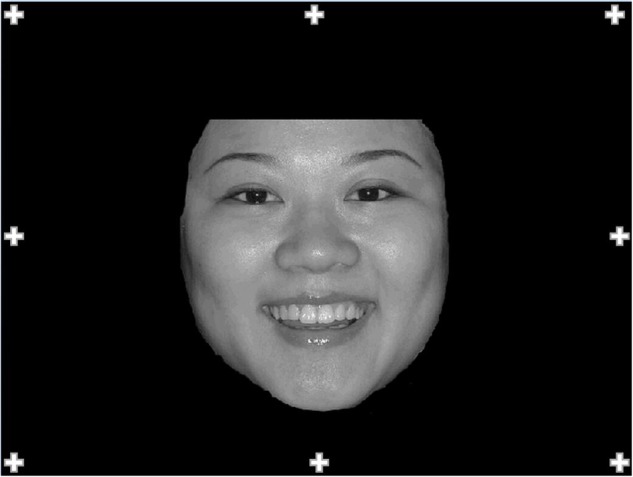
**The location of the eight positions and their relative position on the displayed face for Experiment 2**.

Almost all procedure details in Experiment 2 were the same as Experiment 1 apart from one aspect: a trial in Experiment 2 started with a fixation point appearing no longer in the middle of the screen but instead randomly in one of eight positions around the boundary of the screen for 1000 ms (see **Figure 2**). This method was used for prevent participant’s fixation was guided to the lower half of the face by the fixation point.

### Defining AOIs

The way to define AOI in Experiment 2 was the same as Experiment 1.

### Results

#### Accuracy According to Facial Expression

The detail data for AC in Experiment 2 was presented in the **Table 1**. We used repeated measures ANOVA to test if there were significant differences in AC, with emotion type as the within-subjects factor. The results showed a significant main effect of emotion type, *F*(3,57) = 12.144, *p* < 0.001, *M* = 0.99, *SD* = 0.01 for happy face; *M* = 0.98, *SD* = 0.01 for pace face; *M* = 0.90, *SD* = 0.02 for sad face and *M* = 0.85, *SD* = 0.03 for anger face. A LSD comparison found that the ACs of both happy and peaceful faces were higher than those of the sad and angry faces (happy vs. sad, *p* < 0.01. and angry, *p* < 0.01; peaceful vs. sad, *p* < 0.01; peaceful vs. angry. *p* < 0.01); however, happy vs. peaceful and sad vs. angry did not differ significantly (*p* > 0.05 for both).

#### AOI Analysis

Using the described AOIs, the statistical analysis of the fixation numbers and durations in different AOIs was conducted. Only the right trails were analyzed.

The detail data for fixation in Experiment 2 was presented in the **Table 2**. We used repeated measures ANOVA to test if there was a significant difference in the number of fixations, with type of emotion expressed and AOI as within-subject factors. There was a significant main effect of emotion type, *F*(3,57) = 6.075, *p* < 0.01, *M* = 2.09, *SD* = 1.93 for happy face; *M* = 2.46, *SD* = 1.96 for pace face; *M* = 2.69, *SD* = 2.29 for sad face and *M* = 2.88, *SD* = 2.37 for anger face. LSD comparisons found that the mean number of fixations for the happy face was lower than that of the other emotion faces (happy vs. peaceful, *p* < 0.05; happy vs. sad, *p* < 0.01; happy vs. angry, *p* < 0.05). Furthermore, the number of fixations for the peaceful face was lower than that for the sad face (*p* < 0.05), but did not differ from that of the angry face (*p* > 0.05). Furthermore, the sad and angry faces did not differ from each other (*p* > 0.05). The main effect of AOI was significant, *F*(1,19) = 5.84, *p* < 0.05, *M* = 0.95, *SD* = 1.16 for eye AOI and *M* = 4.11, *SD* = 1.26 for non-eye AOI, indicating that the number of fixations for the eye AOI was lower than that for the non-eye AOI. The interaction between emotion type and AOI was not significant, *F*(3,57) = 2.067, *p* > 0.05.

The detail data for duration in Experiment 2 was recorded in the **Table 3**. We used repeated measures ANOVA to test if there was a significant difference in fixation duration, with emotion type and AOI as the within-subject factors. We found a significant main effect of emotion type, *F*(3,57) = 6.038, *p* < 0.01, *M* = 528.11, *SD* = 474.27 for happy face; *M* = 625.28, *SD* = 497.63 for pace face; *M* = 684.54, *SD* = 574.08 for sad face and *M* = 739.59, *SD* = 577.31 for anger face. LSD comparisons showed that fixation duration was shorter for happy faces compared to sad or angry faces (happy vs. sad, *p* < 0.01; happy vs. angry, *p* < 0.05), but not different from the peaceful face (*p* > 0.05). Furthermore, the fixation duration for the peaceful face was shorter than that of the sad face (*p* < 0.01), but not different from that of the angry face (*p* > 0.05). The fixation durations of the sad and angry faces did not differ from each other (*p* > 0.05). The main effect of AOI was significant, *F*(1,19) = 7.19, *p* < 0.05, *M* = 226.89, *SD* = 266.09 for eye AOI and *M* = 1061.87, *SD* = 287.20 for non-eye AOI. The fixation duration on the upper face was shorter than that of the lower face. The interaction between emotion type and AOI was not significant, *F*(3,57) = 1.261, *p* > 0.05.

## Discussion

In this study, we asked if healthy individuals from Eastern cultures developed a pattern of avoiding eye contact when identifying facial expressions displayed by Eastern faces. To answer this question, we used eye-movement technology and designed two experiments to explore the fixation patterns of Chinese participants when they attempted to identify such expressions.

Experiment 1 demonstrated an opposite pattern compared to those of studies using Western samples (Vassallo et al., 2009)—the number of fixations and fixation durations were less for the upper part of the face (eyes) compared to the lower part of the face (nose and mouth). Experiment 2 repeated this finding and excluded the possibility of fixation point guiding.

This difference in fixation patterns between Eastern and Western participants could be explained by their respective customs during social interactions. In Western culture, direct eye contact during communication shows respect to others and is frequently encouraged (Argyle and Cook, 1976), as evidenced by the American maxim, “Don’t trust a man who won’t look you in the eye.” Therefore, in Western cultures, the eyes are viewed more often than the mouth and nose when gazing at a face. By contrast, in Eastern, due to the etiquette culture, direct eye contact when communicating is considered very rude and should be avoided (Argyle and Cook, 1976). Therefore, people from Eastern cultures view the eyes less often than the mouth and nose. However, our results partly contradict that of Jack et al. (2009), who found a superiority effect for the upper part of the face in participants from Eastern cultures when identifying all seven types of facial expressions (the six basic emotions plus a neutral face). It is possible that the difference in results is due to different AOI sizes. Our AOI for the lower part of the face was much bigger than that for the upper part of a face, which may lead to more fixations on the lower part. However, this was probably not the case, because Jack et al.’s (2009) participants fixated more on the eyes (Jack et al., 2009, Figure 1A, p. 1544), which is independent of the size of the AOI. Another possible reason for the difference was the stimuli used. Jack et al. (2009) used FACS-coded facial expressions (Biehl et al., 1997) as stimuli, which might be considered artificial. Indeed, the authors found superiority of the upper face even for happy faces, which contradicts the results of a study that used CAFPAS (Wang and Luo, 2005), a face database especially design for Chinese. Moreover, a study (Cui et al., 2009) that also used FACS-coded facial expressions did not find a superiority effect of the upper face when gazing at a happy face. Thus, we think the ecological validity of the FACS-coded facial expression is relatively low, leading to unreliable results.

Another interesting question is if this explanation holds true for each type of facial expression. For example, the recognition of a happy expression depends more on the lower portion of the face than the upper portion of the face (Cui et al., 2009). Therefore, our present finding of more and longer fixations for the lower portion of happy faces may mean that the eyes are simply not important for recognizing a happy face, thereby indicating that this was not due to the influence of Chinese culture. In contrast, it has been shown that recognizing a sad face depends more on the upper half of the face (Cui et al., 2009), so our finding of more and longer fixations for the lower portion of sad faces may be influenced by culture. Further research should include more types of facial expressions to ascertain if such eye movement patterns apply to other basic emotions, such as fear or surprise.

Another interesting outcome was that Sui and Ren (2007) found that fixation started at the right eye, while we found that fixation started primarily in the non-eye area; only a few fixations started at the eye, and they were at the left eye. The difference in materials may be one explanation. Although both studies used Asian participants, in the former study (2007), all actors displaying the emotion faces were from Western cultures, while in our study, Eastern actors were used. The in-group advantage (Zhang et al., 2011) in facial expression recognition suggests that there may be different fixation models while identifying native compared with non-native facial expressions. Further research could use eye movement technology to make a direct comparison between in- and out-group emotion faces to test this hypothesis.

## Conclusion

The present experiment found that Chinese people showed a superiority effect of the lower portion of the face while identifying four facial expressions displayed by Chinese faces: happy, peaceful, sad, and angry.

## Author Contributions

MX was responsible for experiment design; data collection; writing; XL was responsible for experiment design, writing and revision; HL was responsible for experiment design and revision; HZ was responsible for writing and revision.

## Conflict of Interest Statement

The authors declare that the research was conducted in the absence of any commercial or financial relationships that could be construed as a potential conflict of interest.

## References

[B1] Argyle M. Cook M. (1976). *Gaze and Mutual Gaze.* Oxford: Cambridge University.

[B2] Biehl M. Matsumoto D. Ekman P. Hearn V. Heider K. Kudoh T. (1997). Matsumoto and Ekman’s Japanese and Caucasian facial expressions of emotion (JACFEE): reliability data and cross-national differences. *J. Nonverbal Behav.* 21 3–21. 10.1023/A:1024902500935

[B3] Blais C. Jack R. E. Scheepers C. Fiset D. Caldara R. (2008). Culture shapes how we look at faces. *PLoS ONE* 3:e3022 10.1371/journal.pone.0003022 PMC2515341 18714387

[B4] Caldara R. Zhou X. Miellet S. (2010). Putting culture under the ‘spotlight’ reveals universal information use for face recognition. *PLoS ONE* 5:e9708 10.1371/journal.pone.0009708 PMC2841167 20305776

[B5] Cui X. Wang Z. Jiang C. Tian B. (2009). Asymmetry of emotional information in upper and lower facial expression. *Psychol. Sci.* 32 1183–1185.

[B6] Emery N. J. (2000). The eyes have it: the neuroethology, function and evolution of social gaze. *Neurosci. Biobehav. Rev.* 24 581–604. 10.1016/S0149-7634(00)00025-7 10940436

[B7] Hampson E. van Anders S. M. Mullin L. (2006). A female advantage in the recognition of emotional facial expressions: test of an evolutionary hypothesis. *Evol. Hum. Behav.* 27 401–416. 10.1016/j.evolhumbehav.2006.05.002

[B8] Horley K. Williams L. M. Gonsalvez C. Gordon E. (2003). Social phobics do not see eye to eye: a visual scanpath study of emotional expression processing. *Anxiety Disord.* 17 33–44. 10.1016/S0887-6185(02)00180-9 12464287

[B9] Horley K. Williams L. M. Gonsalvez C. Gordon E. (2004). Face to face: visual scanpath evidence for abnormal processing of facial expressions in social phobia. *Psychiatry Res.* 127 43–53. 10.1016/j.psychres.2004.02.016 15261704

[B10] Jack R. E. Blais C. Scheepers C. Schyns P. G. Caldara R. (2009). Cultural confusions show that facial expressions are not universal. *Curr. Biol.* 19 1543–1548. 10.1016/j.cub.2009.07.051 19682907

[B11] Liu S. Wang H. Peng K. Zheng X. Liu Z. Xu S. (2013). Cross-cultural research on attention and its implications. *Adv. Psychol. Sci.* 21 37–47. 10.3724/SP.J.1042.2013.00037

[B12] McCarthy A. Lee K. Itakura S. Muir D. W. (2006). Cultural display rules drive eye gaze during thinking. *J. Cross Cult. Psychol.* 37 717–722. 10.1177/0022022106292079 19122788 PMC2613330

[B13] Schimmack U. Oishi S. Diener E. (2002). Cultural influences on the relation between pleasant emotions and unpleasant emotions: Asian dialectic philosophies or individualism-collectivism? *Cogn. Emot.* 16 705–719. 10.1080/02699930143000590

[B14] Schneier F. R. Rodebaugh T. L. Blanco C. Lewin H. Liebowitz M. R. (2011). Fear and avoidance of eye contact in social anxiety disorder. *Compr. Psychiatry* 52 81–87. 10.1016/j.comppsych.2010.04.006 21220069 PMC9731729

[B15] Senju A. Johnson M. H. (2009a). Atypical eye contact in autism: models, mechanisms and development. *Neurosci. Biobehav. Rev.* 33 1204–1214. 10.1016/j.neubiorev.2009.06.001 19538990

[B16] Senju A. Johnson M. H. (2009b). The eye contact effect: mechanisms and development. *Trends Cogn. Sci.* 13 127–134. 10.1016/j.tics.2008.11.009 19217822

[B17] Sui X. Ren Y. (2007). Online processing of facial expression recognition. *Acta Psychol. Sin.* 39 64–70.

[B18] Tan C. B. Y. Stephen I. D. Whitehead R. Sheppard E. (2012). You look familiar: how Malaysian Chinese recognize faces. *PLoS ONE.* 7:e29714 10.1371/journal.pone.0029714 PMC3256166 22253762

[B19] Vassallo S. Cooper S. L. Douglas J. M. (2009). Visual scanning in the recognition of facial affect: Is there an observer sex difference? *J. Vis.* 9 11 1–10. 10.1167/9.3.11 19757950

[B20] Wang Y. Luo Y. (2005). Standardization and assessment of college students‘ facial expression of emotion. *Chin. J. Clin. Psychol.* 13 396–398.

[B21] Zhang Q. Chen J. Yu Q. Xin P. (2011). An in-group advantage in recognizing emotion. *Adv. Psychol. Sci.* 19 209–216.

